# Impact of compliance to oral cysteamine treatment on the costs of Kidney failure in patients with nephropathic cystinosis in the United Kingdom

**DOI:** 10.1186/s12882-023-03392-y

**Published:** 2023-11-29

**Authors:** Seun Lashilola, Weiwei Xu, Khashayar Azimpour, Michael McCarthy, Sara Carlot, David Game, Judith van der Voort

**Affiliations:** 1 Real World Insights, IQVIA, London, UK; 2 Real World Insights, IQVIA, Amsterdam, the Netherlands; 3 Global Health Economics and Outcome Research, Chiesi, Boston, United States; 4 Health Economics and Outcomes Research, MAP Patient Access, Cambridge, UK; 5 Global Rare Diseases Business Unit, Chiesi Farmaceutici S.p.A, Parma, Italy; 6 Department of Nephrology, Guy’s Hospital, London, UK; 7 Department of Paediatric Nephrology, University Hospital of Wales, Cardiff, Wales

**Keywords:** Nephropathic cystinosis, Kidney Failure, Treatment compliance, Delayed-release Cysteamine Bitartrate, Adherence, United Kingdom

## Abstract

**Background:**

Nephropathic Cystinosis (NC), a rare disease characterised by intra-lysosomal accumulation of cystine, results in progressive kidney failure (KF). Compliance to lifelong oral cysteamine, the only therapy, is often compromised. The relationship between compliance and costs of NC has not been previously formally assessed. The present study evaluates the impact of compliance on lifetime (direct) costs of treating KF in NC patients in the United Kingdom.

**Methods:**

A three-state (KF-free, post-KF, death) partitioned survival model was developed for hypothetical ‘Good Compliance’ (GC) and ‘Poor Compliance’ (PC) cohorts. Survival in the KF-free state was determined by a published regression function of composite compliance score (CCS). The CCS is a summation of annual compliance scores (ACS) over treatment duration prior to KF. ACSs are indexed on annual (average) leukocyte cystine levels (LCL). The Poor Compliance cohort was defined to reflect NC patients in a previous study with a mean LCL of 2.35 nmols nmol half-cystine/mg protein over the study period – and an estimated mean ACS of 1.64 over a 13.4 year treatment duration. The Good Compliance cohort was assumed to have an ACS of 2.25 for 21 years. Major KF costs were evaluated – i.e., dialysis, kidney transplants, and subsequent monitoring.

**Results:**

The mean CCS was 47 for the GC and 22 for the PC cohort respectively, corresponding to estimated lifetime KF costs of £92,370 and £117,830 respectively – i.e., a cost saving of £25,460/patient, or £1,005/patient for every 1-unit improvement in CCS.

**Conclusion:**

This analysis indicates that lifetime costs of KF in NC can be reduced through improved treatment compliance with oral cysteamine.

**Supplementary Information:**

The online version contains supplementary material available at 10.1186/s12882-023-03392-y.

## Background

Cystinosis, a rare autosomal recessive disorder, is characterised by intra-lysosomal accumulation of cystine induced by a mutation in the CTNS gene (17p13) that encodes a lysosomal cystine/proton cotransporter: cystinosin [1, 2]. Cystine accumulation leads to cellular damage in different organs and tissues, initially primarily the eyes and kidney, resulting in progressive kidney failure (KF) [1, 3]. Cystinosis is an extremely rare disease, with a global incidence of 1:100,000 to 1: 200,000 live births [4]. Although different phenotypes of the disease may overlap, cystinosis is classified in three clinical forms: nephropathic (early onset infantile or late onset juvenile), intermediate (late onset adolescent), or non-nephropathic ocular (adult or benign), with nephropathic cystinosis (NC) being the most severe and common, affecting ~ 95% of patients [1, 2]. Owing to its rarity, the diagnosis of NC is frequently delayed, imposing a considerable impact on long-term prognosis [3, 5].

As a multisystemic progressive disorder necessitating lifelong medical treatment, NC poses a considerable health and economic burden [6–8]. NC leads to significantly reduced health-related quality-of-life (QoL), and impaired cognitive, behavioural, and school/work functioning [9, 10]. It also leads to a considerable psychological burden and reduced QoL for families and/or caregivers of patients with NC [7]. KF is a key complication of cystinosis along with dehydration and electrolyte imbalance due to renal tubular Fanconi syndrome [11]. For patients with NC, KF may arise as early as 10 years of age [4]. KF due to NC imposes a substantial economic burden on the healthcare system, patients, and caregivers, owing to kidney replacement therapy in the form of long-term dialysis or kidney transplantations, hospitalisations, emergency room visits, and need for medication [12–14].

Lifelong cystine-depleting therapy with oral cysteamine is the only specific targeted therapy available for the management of NC [2, 8, 15]. Early initiation and strict, sustained, long-term compliance to treatment have proven essential for improved kidney outcomes in cystinosis [8, 16, 17]. However, long-term compliance is often compromised due to adverse effects such as persistent body odour, halitosis, vomiting or diarrhoea, and the rigorous dosing regimen – every 6 h (Q6H) [18, 19]. As a result, a delayed-release oral cysteamine formulation was developed and has received marketing authorisation in the US and Europe [20, 21]; it is administered twice daily (every 12 h), avoiding the need for night-time administration, and reducing adverse effects - thus improving barriers to treatment compliance. However, while there are studies indicating high levels of treatment compliance with this formulation [22, 23], long-term real-world evidence is not yet available. Conversely, Nesterova et al. (2015), retrospectively estimated long-term compliance for a cohort of patients treated with (immediate-release) cysteamine at the National Institutes of Health (United States), between 1975 and 2005; they used levels of leucocyte cystine depletion as a proxy for annual compliance [13]. Furthermore, the authors demonstrated a positive linear relationship between compliance levels and age at kidney failure: their analysis indicating that for every year of excellent compliance, nearly 1 year of kidney function was preserved [13]. This study is further discussed in the methods section below.

Utilising the analysis by Nesterova et al. (2015) [13], the present analysis aims to assess the potential relationship between NC treatment compliance and major costs of KF (i.e., dialysis, transplants, and subsequent monitoring) for NC patients, by estimating and comparing the lifetime KF costs for hypothetical ‘Good Compliance’ and ‘Poor Compliance’ cohorts. The present study is conducted from the perspective of a UK healthcare provider.

## Methods

### Overview

In the present analysis, the following steps were taken to estimate the potential relationship between treatment compliance and the lifetime cost of KF:

1) Two hypothetical cohorts ─ Good Compliance and Poor Compliance ─ were defined based on an assumed mean age-at-KF for a representative patient in each respective cohort. The age-at-KF assumptions were based on real-world evidence [13, 17] and clinical expert opinion.

2) For each cohort, the age-at-KF value was used to estimate a composite compliance score (CCS), which in turn was used to estimate an annual compliance score (ACS) – both estimations were based on the model derived by Nesterova et al. (2015) [13].

3) For each cohort, a mean age-at-death was estimated, based on real-world evidence [8] and clinical expert opinion.

4) (A) A survival to kidney failure (SKF) curve was developed to reflect the mean age-at-KF for each cohort. (B) Also, an overall survival (OS) curve was developed to reflect mean age-at-death for each cohort.

5) Using partitioned survival analysis, the years spent in a ‘post-KF’ state (the difference between mean age-at-death and mean age-at-KF), for each cohort, were calculated.

6) The lifetime cost of KF was estimated - by multiplying the number of years spent in post KF by the annual cost of KF.

7) The lifetime cost was adjusted to account for an annual discount rate – to produce discounted lifetime costs of KF for each cohort. This concept of ‘discounting’ is described in a section below.

8) The difference in discounted lifetime KF costs between both cohorts is calculated. Of note, results reflect mean (or per-patient) costs.

The steps outlined above are explained in more detail in the following sections.

### Clinical inputs: estimating mean age-at-KF and mean age-at-death (steps 1–3)

The present analysis utilises the study by Nesterova et al. (2015) investigating the relationship between treatment compliance and age-at-KF (in years) using 30 years of clinical follow-up data for 53 patients with NC followed from birth in the United States [13]. In their study, compliance was defined as a composite score taking into consideration both a patient’s duration of treatment prior to KF (in years) and their annual compliance score (ACS). A patient’s ACS was indexed on a patient’s mean leucocyte cystine level within that annum (henceforth: annual LCL) (Table 1) [13]. A patient’s CCS, therefore, is the sum of a patient’s ACSs over their duration of treatment prior to KF. Nesterova et al. (2015) estimated a linear model with the equation 𝑦 = 0.30𝑥 + 8.82; ${R}^{2}$ = 0.61 (henceforth, Nesterova’s equation), where 𝑥 is a patient’s CCS, and 𝑦 is their age-at-KF [13].

**Table 1:** Compliance index based on patients cystine level, Nesterova et al. (2015)

Compliance score	Cystine level range (nmol half-cystine/mg protein)
$0$	$x \ge 3.0$
$1$	$2.0 \ge x < 3.0$
$2$	$1.0 \ge x < 2.0$
$3$	$x < 1.0$

### Clinical inputs: estimating mean age-at-KF and mean age-at-death (steps 1–3)

Building on Nesterova et al. (2015) [13], the present analysis focuses on patients monitored from birth, starting oral cysteamine treatment at an early age (i.e., before 5 years of age). Of note, Nesterova et al. (2015) [13] do not make explicit the mean age of treatment initiation in their study sample; our assumption of an early age is based on clinical expert advice and the consistency of outcomes with similar studies (Brodin-Sartorius et al. (2012) [8], Emma et al. (2021) [17]) which consider an ‘early treatment’ sample. Importantly, clinical experts interviewed also suggested that such patients best represent the patient population in the UK.

Two hypothetical cohorts – ‘Good Compliance’ and ‘Poor Compliance’ – were defined, and Nesterova’s equation [13] was used to estimate treatment specific CCSs for patients by assuming a mean age-at-KF for each cohort and substituting them into the equation.

#### Estimating mean age-at-KF

The Poor Compliance cohort was intentionally defined to reflect the patients assessed by Nesterova et al. (2015) [13]. Patients in this study were reported as having a mean LCL^1^ of 2.35 (± 0.26) nmols half-cystine/mg protein (henceforth: nmols). Given the definition of optimal cystine control in the literature (a leukocyte cystine level < 1 nmol) [23], and feedback from clinical experts, these patients were deemed to sufficiently reflect a ‘poor compliance’ cohort. Thus, the aim was to model a Poor Compliance cohort that reflected the real world in terms of compliance and, consequently, to use a mean age-at-KF result that was externally valid − indeed, identical to the result reported by Nesterova et al. (2015) [13]. Therefore, the mean CCS applied to the Poor Compliance cohort was calculated based on the reported mean age-at-KF in Nesterova et al. (2015) − a mean age-at-KF of 15.40 years, implying a mean CCS of 21.93 using their equation [13] (Table 2). This CCS also implies a mean ACS of 1.42 (as $1.42\times 15.40=21.93$). In the index (henceforth, Nesterova’s index) provided by Nesterova et al. (2015) (Table 1), an ACS of 1 corresponds to an annual LCL ≥ 2 and < 3; an ACS of 2 corresponds to an annual LCL ≥ 1 and < 2 [13]. Therefore, given the definition of optimal cystine control mentioned above, a mean ACS of 1.42 plausibly reflects a Poor Compliance cohort. Of note, while the mean ACS expresses the average ACS over all the years of a patient’s life until age-at-KF, to judge compliance levels it is more accurate to consider the ACS over the expected treatment period. Based on clinical expert guidance and published literature [17] we assumed that the average age of treatment initiation is ~ 2 years old. Applying this assumption to our previous calculation entails an ACS of 0 over these first 2 years. Thus, the mean ACS over the remaining 13.4 years (the assumed treatment period) is 1.64 (as $1.64\times 13.40=21.93$). This compliance level also plausibly reflects a Poor Compliance cohort. The representative patient in the Poor Compliance cohort was assumed to have this ACS, 1.62, and a treatment duration of 13.4. However, the choice of ACS and treatment duration ultimately do not affect the base case results of this analysis – it is the CCS (which can be obtained with various ACS and treatment duration combinations)^2^ that can be employed to generate the same CCS. Similarly, for the purpose of drawing conclusions, regarding the relationship between compliance and the cost KF, the CCS is sufficient and is the parameter that was ultimately used as a standard for comparison.

**Table 2:** Cohort Definitions

Cohort	Model Input	Value/Definition	Assumption
Poor Compliance	Mean age-at-KF (years)	15.40	Reported value in Nesterova, et al. (2015) [13]
	Composite Compliance Score	21.93	Calculated using Nesterova’s equation [13]: 𝑦 = 0.30𝑥 + 8.82. Where $y = 15.40$
	Treatment duration prior to KF (years)	13.40	Assumption: equal to the mean age-at-KF minus 2 years
	Annual Compliance Score	1.62	Calculated based on treatment duration and CCS: 21.93 ÷ 13.40 = 1.62
	Mean age-at-death (years)	48.77	Derived from survival analysis applied to Kaplan-Meier mortality data provided in Brodin-Sartorius et al. (2012) [8]; validated by clinical experts
Good Compliance	Mean age-at-KF (years)	23.00	Assumption: based on clinical expert guidance
	Composite Compliance Score	47.27	Calculated using Nesterova’s equation [13]: 𝑦 = 0.30𝑥 + 8.82. Where $y = 23.00$
	Treatment duration prior to KF (years)	21.00	Assumption: equal to the mean age-at-KF minus 2 years
	Annual compliance score	2.25	Calculated based on treatment duration and CCS: 47.27 ÷ 21.00 = 2.25
	Mean age-at-death (years)	56.37	Calculated by assuming the difference in mean age-at-KF between both cohorts is equal to the difference in mean age-at-death in both cohorts; validated by clinical experts

LCL: leukocyte cystine level, CCS: Composite Compliance Score, KF: Kidney Failure, ACS: Annual Compliance Score

For the Good Compliance cohort, a mean age-at-KF of 23 years was assumed. Clinical experts suggested that a good level of compliance (i.e., a mean ACS between 2 and 3) would likely result in an age-at-KF value within a range of 18–30 years − particularly given the assumption of early treatment initiation (i.e., < 5 years of age). This is corroborated by the literature. For example, considering the top 10% of CCSs (i.e., the most compliant patients) assessed in Nesterova et al. (2015), the mean age-at-KF observed was approximately 23 years [13]. Although Nesterova et al. (2015) do not make explicit the age at treatment initiation for these patients, it is likely these patients started treatment early; recent data from Emma et al. (2021) make clear that age at renal failure (defined as stage 5 chronic kidney disease) is significantly associated with age at treatment initiation [17, 24]. Furthermore, Emma et al. (2021) also shows a median age of approximately 18 years before renal failure for patients with a mean leucocyte cystine level between 1.2 and 1.8 nmols, irrespective of age at initiation or treatment duration [17]. Given the ‘age-at-KF’ range (18–30 years) proposed, the lower/upper bounds of the range were tested in scenario analyses. Using Nesterova’s equation [13] a mean age-at-KF of 23 years resulted in a CCS of 47.27, and a mean ACS of 2.06 (as $2.06\times 23=47.27$). As with the Poor Compliance cohort, assuming patients start treatment after 2 years, the mean ACS over the assumed treatment period (21 years) is 2.25 (as $2.25\times 21=47.27$). In both cases, the mean ACS plausibly reflects a Good Compliance cohort. The representative patient in the Good Compliance cohort was assumed to have this ACS, 2.25, and a treatment duration of 21 years. Furthermore, in some sensitivity analyses the age-at-KF was analysed as a function of these parameters (ACS and treatment duration) - using Nesterova’s equation [13]. As a result, we could test the sensitivity of results to changes in ACS or treatment duration – indeed we did test the results against alternate ACS assumptions, and indirectly tested the results against alternate treatment duration assumptions by testing alternate age-at-KF assumptions.

In summary, a mean age-at-KF of 23.00 years and 15.40 years was assumed for the Good Compliance and Poor Compliance cohort, respectively (Table 2). Substituting these values into the Nesterova’s Eq. (2015) [13], the resulting mean CCSs were 47.27 in Good Compliance and 21.93 in Poor Compliance (Table 2). The ACSs derived based on the CCSs corroborated the assumption that 23.00 years and 15.40 years were plausible estimates of age-at-KF for patients in Good Compliance and Poor Compliance respectively.

#### Estimating mean age-at-death

It was also necessary to estimate the mean age-at-death for both cohorts – in order to estimate the difference between the mean age-at-death and mean age-at-KF (that is, time spent in a ‘post KF’ state). Ideally, mean age-at-death would have been assumed based on literature regarding the relationship between compliance and age-at-death – as was done for the assumptions of mean age-at-KF. However, an extensive literature search was conducted and found no direct evidence of a relationship between compliance and mean age-at-death. Instead, for both cohorts, mean age-at-death was based on clinical expert guidance regarding the use of mortality data provided in Brodin-Sartorius [8]. The methodology is discussed in more details below. The resulting mean age-at-death was 48.77 years in the Poor Compliance cohort, and 56.37 years in the Good Compliance cohort (Table 2).

### Clinical inputs: Estimating time spent in the Post KF state and associated costs (steps 4–8)

For each cohort, the aim was to use the mean age-at-KF and mean age-at-death values to estimate the time spent in a ‘post KF’ state. The number of years spent in this state would be multiplied by an annual cost of KF to estimate a lifetime KF cost (per-patient). Importantly, ‘discounting’ would be applied in this process to establish a discounted lifetime KF cost for each cohort. Discounting in health economic analysis is a standard technique that aims to reflect the idea that costs and/or health outcomes predicted to occur in the future are usually valued less than present costs and/or health outcomes [25]. It is important to account for discounting particularly when comparing interventions and/or cohorts for which associated costs and/or health outcomes occur at differential times ─ as is the case in the present analysis. Discounting is usually included by estimating the costs (and/or health benefits) incurred in each year (or other defined time interval), over a given time horizon, for a cohort of patients. A discounting factor is applied to each value in the series and aggregated to a give a ‘present value’ of the entire series. The discount factor increases over time, based on an underlying discount rate. National Institute of Health and Care Excellence (NICE) guidelines recommend that costs and health outcomes should be discounted at 3.5% per year [26]. So, for example, £100 incurred in Year 2 would have a present value of £96.62. For Year 11, the present values would be £70.89 [25]. Therefore, in the present analysis, for each cohort, the aim was to estimate the mean lifetime KF cost as the present value of the sum of a series of annual costs incurred in the post KF state ─ accounting for differences in the timings of costs.

#### Partitioned survival model (PSM): structure

**Fig. 1:**
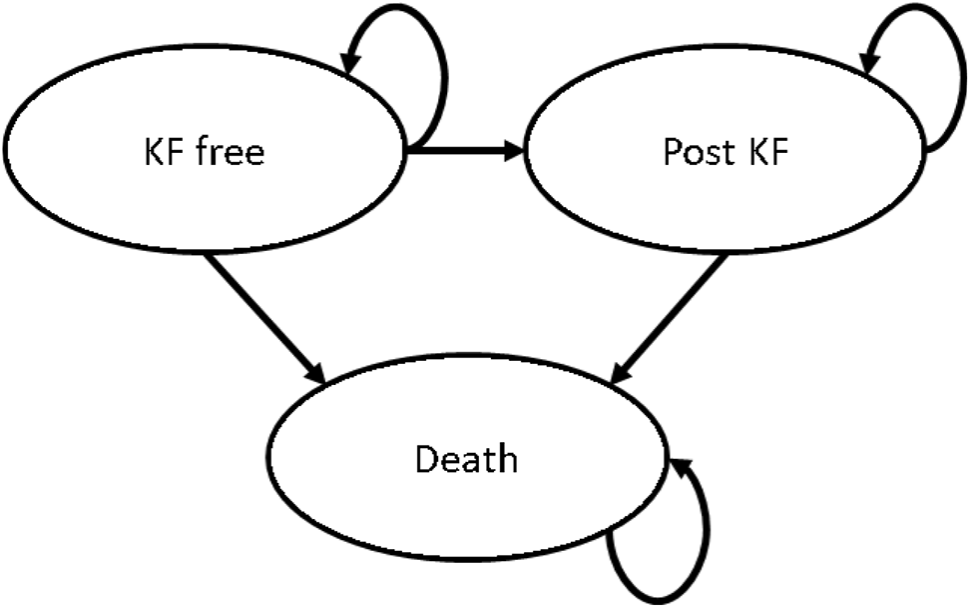
Three primary health states of partitioned survival model. KF: Kidney Failure

Therefore, for each cohort, a partitioned survival model was developed in Microsoft Excel® to model the discounted lifetime costs of KF associated with each cohort. The model included three mutually exclusive health states − ‘KF free’, ‘post-KF’, and death (Fig. 1). One-year cycle lengths, and a ‘lifetime’ time horizon (100 years) were assumed. For each cohort, state membership in any given year was determined by two independently modelled, non-mutually exclusive, survival curves – a survival to kidney-failure (SKF) curve, and overall survival (OS) curve. Patients were assumed to enter the model at birth and, in each year, the probability of reaching KF, or death respectively, for a patient was applied based on the probabilities for an individual of that age. These probabilities (and thus, survival curves) were cohort specific. That is, the SKF curve for each cohort reflected the corresponding, assumed, mean age-at-KF (henceforth, time-to-KF). Similarly, the OS curves reflected the mean age-at-death (henceforth, time-to-death) estimates. The modelled SKF curves were based upon on survival probabilities taken from real world data regarding the time-to-KF for patients with NC [8]. The survival probabilities do not account for other causes of KF (including co-existing diseases) or patients with a subsequent failure [8]. Thus, membership in the post-KF in this analysis was determined by initial KF caused by NC. However, in the present analysis the SKF curves were adjusted by background mortality risks (derived from the modelled OS curves), and the modelled OS curves reflected all-cause mortality for a NC population [8]. Thus, membership in the state of death could account for other causes. Furthermore, in this analysis, only selected direct costs associated with the ‘post-KF’ state were considered: specifically, the costs of providing dialysis and/or kidney transplants (and transplant maintenance) in secondary care. Hospital care related adverse event costs were not explicitly accounted for in this analysis but were partially implicitly incorporated. The discounted lifetime cost of KF accrued over the time spent in the post-KF state was calculated. PSMs are useful for accounting for the differences in underlying probability distributions and this helps to accurately account for discounted costs. In other words, it is inaccurate to simply count the series of discounted annual costs starting from the time at which the mean time-to-KF occurs and counting though the mean number of years spent in the post-KF state. Instead, the method employed through PSMs is to count a series of discounted costs starting from the start of the time horizon (at t = 0) through to the end of the time horizon, accounting for the probability of membership in the ‘KF free’ state, and the probability of membership in the post-KF state. This effectively produces a discounted lifetime cost that is the mean of all possible discounted lifetime costs in a cohort, rather than producing a discounted lifetime cost that is simply a function of the mean time-to-KF and mean time spent in the post-KF state. Costs were valued at 2019 prices based on the latest available National Schedule of National Health Service (NHS) Costs (2019/2020) [27]. Costs were discounted at 3.5%/year in line with guidance from NICE [26]. Half-cycle corrections^3^ were applied to reduce the potential for bias in the cost estimates [28]. For both cohorts, total and disaggregated, per-patient discounted lifetime costs were calculated, and the cost impact associated with ‘good’ compliance evaluated. Furthermore, the resulting relationship between compliance and cost impact was calculated and expressed as a rate.

#### PSM: Reference survival curves

For each cohort, both survival curves modelled (the SKF and OS curve) were generated by modifying a reference SKF curve, and reference OS curve, respectively. The reference curves represent observed survival patterns in the real world; they were derived from a long-term retrospective study (Brodin-Sartorius et al. (2012)) of clinical outcomes in a cohort of adult NC patients (n = 86, 51% male) in France [8]. In this study, patients were followed from diagnosis (mean age, 2.2 years) until adulthood (mean age at last follow up, 26.7 years) [8]. The study provided SKF and OS, Kaplan-Meier (KM) curves stratified by three subgroups: patients who began oral cysteamine treatment before the age of 5 (the ‘under 5’ group), those who began treatment after the age of 5 (the ‘over 5’ group), and those who had received no treatment until they had reached KF (the ‘no treatment’ group) [8]. Only the KM curves representing the ‘under 5’ subgroup (n = 40), were used to generate the reference curves applied, in the base case analysis of the present study. This is consistent with the assumption that this population represents most patients in the UK.

To model survival over the (lifetime) time horizon in the present analysis ─ beyond the study duration in Brodin Sartorius et al. (2012) [8] ─ a statistical analysis was performed to find a parametric function that best fit the KM data. As individual patient data (IPD) were not available for analysis, pseudo IPD were derived from the KM curves – by digitising the curves and applying the Guyot algorithm [29]. In line with NICE guidance, parametric survival curves (exponential, Weibull, log-normal, log-logistic, Gompertz, gamma, and generalised gamma distributions) were fitted to the pseudo IPD and then extrapolated over the model’s time horizon [30]. Following visual inspection and statistical testing (Akaike information criterion, Bayesian information criterion) the parametric distribution selected for both curves was the log-normal distribution (Supplementary Figs. 1–3; Supplementary Tables  1–3).

However, for OS, although the log-normal extrapolation had the best statistical fit, there was high uncertainty regarding extrapolations due to a significant loss to follow up after 20 years of age (i.e., death is an event that manifests later in the NC disease process). Therefore, model results were tested against all OS extrapolations to observe the sensitivity of results against these assumptions. In addition, data from the ‘over 5’ subgroup was used in a scenario analysis, for which the log-normal distribution was also deemed best-fitting. All curves chosen were validated by clinical experts.

The chosen parametric extrapolations were applied in this analysis with the assumption that risk (of KF and death) estimates are independent of each other. Therefore, for both cohorts, the resulting modelled SKF curve was adjusted by the mortality risks provided by the modelled OS curve.

#### PSM: Modelled SKF curves

In the base case, for both cohorts, the reference SKF curve was modified to reflect the assumed mean time-to-KF values [13] ─ the resulting curves are henceforth referred to as the ‘modelled’ survival curves. This was achieved by deriving the hazard ratio (HR) necessary to produce the assumed mean time-to-KF, and adjusting the hazard rate in the survival function equation for the reference SKF by this HR. Table 3 provides the HRs applied and corresponding resulting mean time-to-KF values. The resulting (modelled) curves were validated by clinical experts.

**Table 3:** Parameters defining reference (log-normal) curves, and associated statistics

Cohort	Event	Mean log (3.d.p.)	Standard deviation log (3.d.p.)	Hazard ratio (2.d.p.)	Median age of onset^a^	Mean age of onset (2.d.p.)
Reference case^b^	KF	2.684	0.439	1.00	15.00	15.98
	Mortality	3.826	0.362	1.00	46.00	48.77
Poor compliance	KF	2.684	0.439	1.01	15.00	15.40
	Mortality	3.826	0.362	1.00	46.00	48.77
Good compliance^ce^	KF	3.062	0.439	0.88	22.00	23.00
	Mortality	3.978	0.362	0.96	54.00	56.37

(a) Nearest whole year, (b) Based on Brodin-Sartorius, (c) KF Hazard Ratio (HR) back-calculated using mean age-at-KF from regression, (d) Mortality HR assumed equal to reference, (e) Mortality HR based on expert opinion

Please also note that ‘age of onset’ = time-to-KF (TTK) or time-to-death (TTD)

d.p.: decimal places, KF: Kidney Failure

Of note, the reference SKF curve, derived from the study by Brodin-Sartorius et al. (2012) [8], was also suitable for representing disease progression for Poor Compliance patients; the data informing the reference curve represents patients in whom 71.8% had a mean leukocyte cystine level > 2 nmols (2 ≤ 34.6% < 3 nmols) [8]. However, the mean time-to-KF data provided by Nesterova et al. (2015) is also correlated with observed compliance, and forms the basis of the linear model applied in the current analysis [13]. For this reason, the modelled Poor Compliance SKF curve reflected the data provided in Nesterova et al. (2015) [13]. However, the reference SKF curve was used to validate the modelled SKF curve in a scenario analysis.

#### PSM: Modelled OS curves

In the Poor Compliance cohort, the reference OS curve derived from Brodin-Sartorius et al. (2012) was used to model disease progression (i.e., the HR was assumed to be 1) [8]. The resulting mean time-to-death was 48.77 (as stated in a previous section). This was considered a reasonable approach because the data informing the reference OS curve represents a population who meet the definition of ‘poor compliance’ in this analysis ─ both the reference SKF curve OS curves are informed by the same population [8]. Furthermore, the resulting mean time-to-death was validated by clinical experts as being clinically plausible. In the Good Compliance cohort, the modelled curve was generated by applying a HR (Table 3) to the reference curve that would result in the difference between mean time-to-death for the Good Compliance and Poor Compliance cohorts being equal to the difference in mean time-to-KF for both cohorts. This resulted in a mean time-to-death of 56.37 (the value stated previously). Clinical experts consulted suggested that this assumption aligned with their observations in the clinical setting and supported the argument that the beneficial impact of compliance in terms of delayed SKF should also be reflected in OS. However, given the uncertainty, sensitivity analyses were conducted to validate the modelled Good Compliance OS curve ─ by making both conservative and optimistic assumptions regarding the curve.

### Cost inputs

The cost of KF was applied in terms of an annual cost. This annual cost was generated through a micro-costing approach in which specific costs were applied to the different stages of a KF model: (a) the waiting list for a transplant; (b) undergoing first transplant; (c) the period between first transplant and transplant failure; (d) undergoing second transplant (the representative patient was assumed to undergo 2 transplants; clinical experts suggested that this is the median number of transplants over a patient’s lifetime); and (e) the period between the second transplant and transplant failure. The specific costs applied were: (a) the monthly cost of dialysis, (b) the initial (first year) annual cost of a transplant, and (c) the subsequent (year 2 and onwards) monthly costs of maintenance post-transplant. It was assumed that these costs would be incurred, in the relevant stages, in the ‘post KF’ state. Therefore, for each cohort, the number of months spent in the ‘post KF’ state was calculated (33.37 years) and used to generate a survival curve, with an assumed exponential distribution, representing patient progression through this state. Estimates of the expected time on the transplant waiting list (9.04 months), and time to transplant rejection/failure (20.62 years) (Table 4) in the real world were applied to these curves to evaluate the mean number of months spent in each of the KF model stages (Table 5). These duration values were multiplied by the relevant unit costs described above to arrive at a mean total cost of KF. These costs were converted into an annual cost of £10,329.65 for patients in both cohorts (Tables 4 and 5).

**Table 4:** Resource Inputs and Cost Inputs for post KF management (micro-costing)

Input	Value	Source
% of patients with a living donor transplant	62.34%*	UK Renal Registry 23rd Annual Report [31], (validated by clinical expert as applicable to English setting)
% of patients with a deceased donor transplant	37.66%*	
Time on waiting list for transplant (months, range): living donor	0 (0–6) ^†^	National Health Service (NHS) England, 2022 [32],
Time on waiting list for transplant (months, range): deceased donor	24 (24–36) ^†^	
Time on waiting list for transplant (months): value used in model	9.04	Calculation: weighted average using living donor % (62.34%) and deceased donor % (37.66%) as weights (validated by clinical expert as applicable to English setting)
Time to transplant rejection or failure (years, range): living donor	22.50 (20.00–25.00) ^‡^	National Health Service (NHS) England, 2022 [32],
Time to transplant rejection or failure (years, range): deceased donor	17.50 (15.00–20.00) ^‡^	
Time to transplant rejection or failure (years): value used in model	20.62	Calculation: weighted average using living donor % (62.34%) and deceased donor % (37.66%) as weights (validated by clinical expert as applicable to English setting)
% on haemodialysis	88%	UK Renal Registry 23rd Annual Report [31], (validated by clinical expert as applicable to English setting)
% on peritoneal dialysis	12%	
Median number of transplants	2	Clinical expert advice
Haemodialysis cost (per month)	£ 2160.78	Weighted average of NHS Reference Costs 2019/20 [33]
Peritoneal dialysis (per month)	£ 2,413.58	Weighted average of NHS Reference Costs 2019/20 [33]
Dialysis cost: value used in model	£ 2,192.12	Calculation: weighted average using % on haemodialysis (88%) and % on peritoneal dialysis (12%) as weights (validated by clinical expert as applicable to English setting)
Kidney transplant – first year (per year)	£ 28,526.52	Kent et al. inflated to 2019/20 [27]
Kidney transplant – subsequent years (per month)	£ 110.93	Kent et al. inflated to 2019/20 [27]
Calculated annual cost inputs based on micro-costing calculations		
Good Compliance	£10,329.65	NA, calculation
Poor compliance	£10,329.65	NA, calculation

KF: Kidney Failure, NA; Not Applicable, NHS: National Health service

*These values were based on the UK’ under 16’ population undergoing renal replacement therapy and were scaled up from their original values: 29% and 48% for % with a living donor and deceased donor, respectively

*These values were based on the UK’ under 16’ population undergoing renal replacement therapy and were scaled up from their original values: 29% and 48% for % with a living donor and deceased donor, respectively

† For patients with a living donor transplant, the lower bound provided by the NHS is 3 months, but clinical experts advised that, for this population, it is 0 months in practice as patients are generally treated pre-emptively. For both types of patients, clinical experts suggested the applying the lower bound in the base case

‡ The ranges were provided by the NHS and the mid-point was assumed in both cases

**Table 5:** Results of micro costing calculations*

Stage	Time spent (months) in each stage: unadjusted	Time spent (months) in each stage: adjusted by probability of survival during that stage	Cost
Pre-transplant dialysis^†^	9.04	8.91	£585.33
First transplant^‡^	NA	0.98	£835.82
Post first transplant maintenance^§^	247.40	180.47	£561.41
First failed transplant dialysis^†^	9.04	4.70	£308.87
s transplant^‡^	NA	0.52	£441.04
Post second transplant maintenance^§^	247.40	95.23	£296.25
s failed transplant dialysis^¶^	NA	111.15	£7,300.93
Total	NA	400.46^#^	£10,329.65

KF: Kidney Failure, NA; Not Applicable, NHS: National Health service

* The probability of survival per month within the post-KF state was estimated by assuming an exponential distribution of survival probabilities based on the mean time spent in the post-KF state (i.e., 33.37 years; equivalent to ~ 400.46 months). Costs were calculated by considering an average patient’s pathway through the consecutive stages. I.e., first through the “pre-transplant dialysis” (waiting list) phase, and then into their first transplant, and then into the maintenance (time to transplant rejection) phase and so on. The amount of time assigned to each stage was taken from real world data (2nd column) subsequently adjusted by the probabilities of survival during the relevant stage (3rd column), and then multiplied by the relevant cost associated with that phase (4th column)

† The time spent in this stage is based on the time spent on the waiting list for a transplant (Table 4). The unit cost applied here is the monthly cost of dialysis (Table 4), £ 2,192.12

‡ The value in the 3rd column of this row does not represent the *time* spent undergoing a transplant (this unspecified time is assumed to be absorbed within the subsequent post, transplant stage). Rather it represents the probability of undergoing a *transplant at that time*. This probability is applied to the full annual cost of a kidney transplant in the first year (Table 4), £ 28,526.52

§ The time spent in this stage is based on the time to transplant rejection or failure (Table 4). It is provided in years (20.62) in Table 4 and converted to months (247.40) here. The unit cost applied here is the subsequent monthly cost of kidney transplant (i.e., maintenance costs), £ 110.93, (Table 4). The unit cost in the first transplant maintenance stage is multiplied by 168.90 months, not 180.47 months, because the cost in the initial 12-month period in this stage (11.57 months accounting for survival probabilities) is accounted for by the annual cost of a transplant. Similarly, the unit cost in the second transplant maintenance stage is multiplied by 89.12 not 95.23 because the cost in the initial 12-month period (6.11 months accounting for survival probabilities) is accounted for by the annual cost of a transplant

¶ As the average patient undergoes 2 transplants, the time spent in this stage is the remaining time in the post-KF state post rejection/failure of the 2nd transplant. Therefore, there is no “unadjusted” value here - the number of months spent in this stage must be directly calculated

# This sum does not account for the values in the “first transplant”, “second transplant”, or “third transplant” rows as these values represent probabilities rather than time

The expected time on the transplant waiting list was a weighted average with weights corresponding to the prevalent proportion of paediatric (16 years old and under) patients receiving a transplant from a living donor (37.66%) or deceased donor (62.34%) in the UK – these proportions were derived from the UK Renal Registry 23rd annual report, published in 2021 [31]^4^. Based on clinical expert guidance and data provided by the NHS [32], the corresponding wating times were 0 months for patients with a living donor (as these transplants are generally conducted pre-emptively) and 24 months for patients with a deceased donor, resulting in a weighted average waiting time of 9.04 months (Table 4). Similarly, the expected time to transplant rejection/failure was a weighted average (20.62 years) calculated based on the same weights (37.66% for deceased donors, and 62.34% for deceased living donor), corresponding to graft failure times of 17.50 years and 22.50 years for deceased and living donors respectively (Table 4).

**Table 6:** Discounted lifetime kidney failure costs

	Good Compliance	Poor Compliance	Difference
**Dialysis**			
Pre-transplant dialysis	£5,234	£6,677	-£1,443
First failed transplant dialysis	£2,762	£3,523	-£761
s failed transplant dialysis	£65,287	£83,282	-£17,995
**Total dialysis cost**	**£73,283**	**£93,482**	**-£20,199**
**Transplant**			
First transplant	£7,474	£9,534	-£2,060
s transplant	£3,944	£5,031	-£1,087
**Total transplant cost**	**£11,418**	**£14,565**	**-£3,147**
**Transplant maintenance**			
Post first transplant maintenance	£5,020	£6,404	-£1,384
Post second transplant maintenance	£2,649	£3,379	-£730
**Total transplant maintenance**	**£7,669**	**£9,783**	**-£2,114**
**Total cost**	**£92,370**	**£117,830**	**-£25,460**

To generate the unit costs of transplants, data were derived from a study by Kent et al. (2015) [27]. This is a study of the annual costs of hospital care for patients with chronic kidney disease in the UK using prospective data from the Study of Heart and Renal Protection trial (n = 7246) [34]. The study provided an estimate of annual direct healthcare costs (hospital admissions and outpatient/day-case attendances) in the first year, post kidney transplant, and for subsequent years of kidney transplant care. Of note, hospital care related adverse events were incorporated into the costs provided by Kent et al. (2015) but disaggregated costs were not provided. These figures were provided in 2010/11 prices and inflated to 2019/20 prices using the hospital & community health services index, and the NHS cost inflation index, provided by the Personal Social Services Research Unit in the UK. In the current analysis, the annual cost for subsequent years was converted into months, whereas all patients undergoing a transplant were assumed to incur the full annual cost in the first year, post-transplant, given that this encompassed the cost of a transplant.

The cost of dialysis was split into two elements: the cost of haemodialysis and cost of peritoneal dialysis. Both were calculated using weighted averages of paediatric and adult dialysis annual costs from the National Schedule of NHS Costs 2019/20 [33] (Supplementary Tables  4–5). They were converted to monthly costs based on the assumption that haemodialysis is required three times per week whilst peritoneal dialysis is conducted daily (Table4) – both assumptions were verified by clinical experts. To develop a mean monthly cost relevant to the UK setting, the distribution of patients across the two modalities was estimated. UK-specific data regarding the prevalence of patients on dialysis and the breakdown by modality were sourced from the UK Renal Registry 23rd annual report [31]. The numbers were converted into proportions: 88% for haemodialysis and 12% for peritoneal dialysis (Table 4).

### Sensitivity analysis

Sensitivity analyses were conducted to test the robustness of base case results against alternative assumptions regarding key and/or uncertain parameters. Most of the alternative scenarios were specific to the Good Compliance cohort. Scenarios included:

1) Alternate assumptions regarding cystine control (or ACS) in Good Compliance ─ assuming an ‘excellent’ annual LCL (i.e., ≥ 0 and < 1nmols), corresponding to a mean ACS of 3.

2) Alternate assumptions regarding mean age-at-KF for the Good Compliance cohort – testing the upper (30 years) and lower (18 years) bound.

3) Alternate assumptions regarding mean time-to-death with Good Compliance ─ increasing (decreasing) the HR applied to the OS curve by + 2.5% (-2.5%): resulting in a time mean time-to-death of 51.43 years (61.85 years).

4) Alternate assumptions regarding time on the transplant waiting list ─ assuming the lower (0 months) and upper bound (24 months).

5) Alternate assumptions regarding time to transplant rejection/failure ─ assuming the lower (17.50 years) and upper bound (22.50 years).

6) Decreasing the discount-rate to 1.5% in line with the NICE recommendations for sensitivity analyses. [35.

7) Assuming the reference SKF curve represents the Poor Compliance cohort − that is, applying a HR of 1; this results in mean-age-at-KF of 15.98 years.

8) Assuming the representative patient in either cohort has 3 transplants instead of 2.

Separately, the sensitivity of results to the choice of parametric models of OS was also tested.

## Results

### Base case results

Overall, the lifetime per-patient KF costs for the Good Compliance cohort was £92,370 as compared to £117,830 for the Poor Compliance cohort, resulting in a cost saving of £25,460 (Table 7)^5^. Dividing this cost saving by the difference between the CCS of each cohort (25.34) suggests that each improvement of one unit of CCS (which is also a one-unit improvement of ACS - which roughly corresponds to a decrease in annual LCL of 1 nmol) gives a lifetime cost saving of £1,005. In both arms, the primary contributor to the KF cost was the cost of dialysis. In the Good Compliance cohort, the lifetime dialysis cost was £73,283, comprising of pre-transplant dialysis (£5,234), dialysis after the first failed transplant (£2,762), and dialysis after the second failed transplant (£65,287). The lifetime dialysis cost for the Poor Compliance cohort was £93,482, involving pre-transplant dialysis (£6,677), first failed transplant dialysis (£3,523), and second failed transplant dialysis (£83,282). The resulting cost saving associated with dialysis was £20,199 over a lifetime. The lifetime overall costs of transplants were £11,418 and £14,565, while the lifetime overall costs of transplant maintenance were £7,669 and £9,783 for the Good Compliance and Poor Compliance cohort, respectively. This resulted in a cost saving for patients with Good Compliance, in terms of transplants (£3,147) and transplant maintenance (£2,114). Table 7 provides a summary of base case results.

**Table 7:** Sensitivity analysis results

Scenario	Unit	Base case	Scenario	Result	Impact on results
Base case	-	-	-	-£25,460	-
OS HR (-2.5%)	-	0.96	0.94	-£7,047	£18,413
Mean age-at-KF (30)	Years	23.00	30.00	-£43,221	-£17,761
OS HR (+ 2.5%)	-	0.96	0.99	-£41,989	-£16,529
Mean age-at-KF (18)	Years	19.50	18.00	-£9,551	£15,910
Annual compliance score: 3	ACS	2.25	3.00	-£37,950	-£12,490
Number of transplants	-	2.00	3.00	-£18,261	£7,200
Applying a HR of 1 to SKF curve in Poor Compliance	-	1.01	1.00	-£20,724	£4,736
Graft failure time (17.50 years)	Years	20.62	17.50	-£29,114	-£3,653
Waiting list for transplant time (36 months)	Months	9.04	36.00	-£28,856	-£3,396
Discount rate (1.5%)	%	0.04	0.02	-£22,188	£3,272
Graft failure time (22.50 years)	Years	20.62	22.50	-£23,500	£1,961
Waiting list for transplant time (0 months)	Months	9.04	0.00	-£24,581	£879

KF: Kidney Failure, HR: Hazard ratio, TTD: Time-to-death

### Sensitivity analysis results

The results of the scenario analyses are presented in Table 6. Model results were most sensitive to alternate assumptions regarding mean time-to- KF, time-to-death, and ACS in Good Compliance. Increasing the mean age-at-KF to 30 years resulted in the largest cost saving (£43,221) – that is, a cost saving £17,761 more than the base-case cost saving (£25,460). Decreasing the mean age-at-KF to 18 years resulted in a cost saving of £9,551. The scenario analysis in which the representative patient was assumed to undergo 3 (rather than 2) transplants resulted in a cost saving of 18,261 (Table 6). Decreasing the HR applied to OS by 2.5% (i.e., increasing the mean time-to-death to 61.85 years) resulted in the smallest cost saving (£7,047), whilst increasing the HR by 2.5% (i.e., decreasing the mean time-to-death to 51.45 years) resulted in a cost saving of £41,989. Assuming an ACS of 3 (i.e., an ‘excellent’ annual LCL: ≥ 0 and < 1nmols) resulted in a cost saving of £37,950. Results were generally insensitive to all other tested scenarios.

Testing results against the different parametric models of OS showed that the results were generally relatively insensitive to the choice of parametric model (Table 8). Of note, using the log-normal function for the ‘over 5’ group resulted in the cost saving of £20,229 – similar to the base case result (£25,460 cost saving) using the log-normal function for the ‘under 5’ group.

**Table 8:** Using different parametric fittings to OS data

Parametric function	Good Compliance	Poor Compliance	Difference
Base case (log normal fitted to ‘under 5’ OS data)	£92,370	£117,830	-£25,460
Log normal curve fitted to ‘over 5’ OS data	£70,053	£90,345	-£20,291
Exponential curve fitted to ‘under 5’ OS data	£216,210	£258,984	-£42,774
Log-logistic curve fitted to ‘under 5’ OS data	£80,823	£102,798	-£21,975
Gamma curve fitted to ‘under 5’ OS data	£81,534	£103,878	-£22,344
Gompertz curve fitted to ‘under 5’ OS data	£57,324	£71,246	-£13,922
Weibull curve fitted to ‘under 5’ OS data	£68,214	£86,110	-£17,896
Generalised gamma curve fitted to ‘under 5’ OS data	£140,475	£206,945	-£66,470

OS: Overall Survival,

## Discussion

In the present analysis, ‘Poor Compliance’ reflects patients assessed by Nesterova et al. (2015) [13] with an estimated ACS of 1.62 (and an observed mean LCL of 2.35); Good Compliance assumes an ACS of 2.25. Given these definitions, the present analysis suggests that there is a discounted cost saving of ~£25,000 over a patient’s lifetime with good treatment compliance relative to poor treatment compliance when considering KF costs only. This is because ‘good’ compliance to treatment results in a slowed annual incidence of KF which, in turn, results in prolonged kidney survival (and overall survival). Therefore, the costs incurred for these patients, on average, are discounted more heavily than costs incurred in Poor Compliance. Indeed, the study reflected the assumption that the undiscounted lifetime cost of KF in each cohort were equal – as the lifetime probability of KF was modelled as 100% for both cohorts and the time spent in the post-KF state was assumed to be identical in both cohorts. Thus, the analysis shows the difference in costs resulting solely from a delay in KF, rather than an avoidance of KF or a difference in the time spent in the post-KF state. The cost saving is largely driven by the difference in the discounted lifetime cost of dialysis between cohorts, with the cost saving associated with dialysis in Good Compliance accounting for ~ 80% of the total cost saving (Table 7) ─ the lifetime cost of dialysis also accounts for ~ 80% of the lifetime cost of KF in each cohort. The bulk (~ 89%) of the cost of dialysis, in both cohorts, is incurred in the “second failed transplant dialysis” period (Table 7). This reflects the fact that the annual cost applied to time spent in the post-KF state is driven by the cost in this period (Table 5). This is consistent with the fact of the relatively high monthly cost of dialysis (Table 7) and the relatively long amount of time spent in this period (Table 5) – as no further transplants are assumed and patients remain in this period until death. Decreasing the transplant survival time (to 17.5 years from 20.62 years) resulted in an increased cost saving (Table 6) of ~ 15%. This is mainly because the shorter time to transplant failure increases the time (and thus cost) spent on dialysis after the second transplant failure. This results in a total cost increase across both arms by ~ 15%: hence the increase in the cost saving. Increasing the transplant waiting time (to 36 months from 9.04 months) increased the total cost saving (by ~ 14%) (Table 6), primarily because it increased the time spent on pre transplant dialysis – both before the 1st and 2nd transplant. The scenario analysis in which 3 (rather than 2) transplants were assumed resulted in a decrease in cost savings from -£25,460 to -£18,261 (Table 6). This is largely due to the decrease in lifetime KF costs in both arms, resulting from less time spent on dialysis (124.8 months in the base case vs. 74.4 months in the scenario analysis). Supplementary Table  6 provides a breakdown of the time and cost results in this scenario.

To our knowledge, this is the first study investigating the impact of compliance to oral cysteamine treatment on the costs of KF, one of the most serious comorbidities associated with NC. Due to a lack of real-world evidence regarding the explicit association between compliance and costs, a modelling approach was adopted. However, the present analysis is informed by real-world evidence regarding the clinical outcomes modelled, and the latest available published data regarding cost and resource-utilisation inputs. The use of the CCS proxy measure and regression analysis provided in Nesterova et al. (2015), is particularly useful. It allows for an accurate reflection of the long-term association between compliance and KF without requiring either directly reported compliance data, or granular data [13]. That is, it allows for making credible hypotheses about outcomes based on assumed mean levels of leukocyte cystine over time, as is done in the present analysis. Furthermore, a recent study by Emma et al. (2021), provides long-term kidney survival data for large cohorts of NC patients defined similarly (according to leukocyte cystine levels) to those modelled in the present analysis [17]. This study validates key clinical inputs and assumptions in the present analysis as it confirms the mean time-to KF assumptions for both cohorts in the present analysis, and makes clear that the risk of renal failure significantly increases with mean leucocyte cystines levels.

The limitations of this study primarily center around uncertainty regarding inputs and assumptions resulting from limited available evidence from literature, and the required synthesis of data from different sources. As already discussed, there was uncertainty surrounding the validity of the reference OS curve. In this case, the curve chosen was statistically best fitting, produced similar results to the best fitting curve for the ‘over 5’ subgroup data (Table 8), and was validated by clinical experts after visual inspection − in terms of the mean, and median, time-to-death produced. Furthermore, given the lack of data regarding a direct association between compliance and mortality, HRs were chosen to reflect the assumption that the difference in mean time-to-death between both cohorts was identical to the difference in mean time-to-KF between both cohorts. The uncertainty associated with this assumption should be noted; clinical experts explained that factors affecting the post-transplant mortality risks in cystinosis make unclear the appropriate assumption. For example, it could be assumed that, post-transplant, the relative mortality risk in the Poor Compliance arm should be greater reflecting a higher co-morbidity burden (from extra-renal cystinosis complications) owing to a history of poor compliance. Conversely, the higher mean age at KF (and therefore transplant) with good compliance may mean that the co-morbidity burden is not significantly different across arms at the time of transplant; furthermore clinical experts explained that the most significant factors in post-transplant-mortality in cystinosis are generally not cystinosis-related and are positively correlated with age. For this reason, scenario analyses varying the chosen HR for the Good Compliance OS curve were conducted. Increasing (decreasing) the HR applied to the OS curve by + 2.5% (-2.5%) resulted in a mean time-to-death of 51.43 years (61.85 years) compared to 56.37 years in the base case in the good compliance arm. The results were highly sensitive to the scenarios, resulting in a cost saving of £41,989 (with an increased HR) and £7,047 (with a decreased HR) compared to the base case cost saving (£25,460). Therefore results should be interpreted with caution. However, the base case assumption was considered the most appropriate given the lack of direct evidence regarding the relationship between compliance and mortality, or evidence that would allow for quantifying the degree to which the difference in mean-time-to death should differ from the difference in mean-time-to KF – and given that this assumption resulted in a mean time-to-death with good compliance (56.37 years) arm that agrees with available evidence both in terms of published data [8, 36, 37] and expert opinion which suggests that that the median overall survival in cystinosis, with proper treatment and early diagnosis, is approximately 50 years.

Another key assumption, in the base case, is that the shape of the modelled survival curves in both cohorts (except for the Poor Compliance OS curve) are the same as their respective reference curve. In other words, it is assumed that if the risk of reaching KF in the modelled Good Compliance SKF curve at time $t$ is twice the risk of reaching KF in the reference SKF curve at time $t$, then it will be twice the risk at all other time points. This assumption had to be made to modify the reference curves ─ that is, the observed survival curves, with established risk profiles (shapes), provided by Brodin-Sartorius et al. (2012) [8]. Clinical experts consulted suggested that, with regards to the Good Compliance OS curve, the shape of the curve might differ to the reference curve. They suggested that improved compliance would result in a short-term risk of death less than currently modelled, and the long-term risk of death is (potentially) greater than currently modelled. This would affect resulted discounted costs, although the direction of the impact is unclear. However, clinical experts also agreed that the choice of the Good Compliance OS curve was the most appropriate choice given the options available in terms of parametric functions. For all other modelled curves, clinical experts validated the curves chosen without objection after visual inspection (in terms of the mean, median, and curve shape).

Nevertheless, given the high result sensitivity to assumptions regarding the OS curves (Tables 6 and 8), this is an area that calls for further research. There is a need for robust long-term mortality data for this specific population of patients with early treatment initiation, and a need for data regarding the association between compliance and mortality generally. Similarly, although the CCS measure is a good proxy for long-term treatment compliance, the gold standard would be to use long-term compliance or adherence data that is directly measured and explicitly associated with relevant survival data. This calls for further research.

Of note, the studies utilised did not analyse a UK population, resulting in uncertainty regarding the generalizability of these results to the UK population. However, clinical experts validated the clinical and resource-use inputs in this analysis as being representative of the UK population. Conversely, the use of UK resource and cost data limit the generalisability of the findings outside of the UK.

Lastly, in this analysis, the mean age-at-KF in the Good compliance cohort ideally would have been strictly predicted by CCS. However, the CCS (and resulting age-at-KF) require a ‘treatment duration prior to KF’ component, for which there was no real-world data to inform. Therefore, the most reasonable assumption regarding this input was one that resulted in a mean age-at-KF similar to results observed in the real-world given a similar annual LCL in Good Compliance – and ratified by clinical experts. Consequently, the mean age-at-KF was chosen rather than predicted. Irrespective, this analysis still fulfils its purpose as an investigation of the impact on lifetime costs resulting from improved compliance. Of note, results were relatively sensitive to scenarios using a lower and upper bound time-to-KF value (Table 6). However, a cost saving was maintained in both scenarios; thus, this analysis can at least provide the range of expected cost savings resulting from improved compliance.

This analysis can be extended in numerous ways. Firstly, given data scarcity, the focus of this analysis was only on one major complication of NC (kidney failure). However, as NC is a disease affecting multiple organ systems [4], the analysis could be extended to account for other major extra-renal complications such as: growth retardation, hypothyroidism, and diabetes mellitus amongst others [1, 8, 17]. Similarly, given the focus of this study, other relevant health care resource costs (e.g., costs relating to chronic kidney disease prior to reaching KF) were not accounted for. Indirect non-medical costs which may be substantial in NC could be incorporated ─ for example, productivity losses for carers may be substantial when caring for children with a lifelong condition such as NC [6, 7]. Also, as this analysis focused only on cost impacts, it did not account for quality-of-life impacts. A cost-utility analysis capturing both the cost impacts and quality of life impacts would provide a more holistic assessment of the true impact of improved compliance in nephropathic cystinosis.

Furthermore, this analysis considers patients diagnosed from birth and initiating treatment shortly thereafter (i.e., before the age of 5). However, there is evidence to show an independent and significant positive relationship between age at treatment initiation and kidney and overall survival [8, 17, 24]. Therefore, this analysis could be extended such that results are stratified not only by compliance levels but also by age at treatment initiation.

This analysis was motivated by early evidence indicating high levels of compliance with the delayed-release formulation of oral cysteamine. This evidence is important in this context as, to date, optimal disease control, an indicator of compliance, has not been demonstrated with the use of immediate-release oral cysteamine [23]. However long-term real-world data regarding treatment compliance with the delayed-release formulation is not yet available. Although the different compliance levels for the hypothetical cohorts in this analysis are not associated with specific treatments, the present analysis provides insight into the potential cost impact, in terms of major health care resource savings, if patients treated with the delayed-release formulation do indeed fall within the definition of ‘Good Compliance’.

## Conclusion

These study results show that improved compliance in patients with NC could result in discounted KF lifetime cost savings of approximately £25,000/patient in the UK. Of note, this study focused solely on the cost of one major NC complication (kidney failure); further research needs to be done to elicit the cost, and quality of life, impact accounting for other complications of NC. This analysis is also subject to some uncertainties, resulting from the absence of long-term real-world data on the compliance/clinical outcome relationship; further research is needed in these areas.

### Electronic supplementary material

Below is the link to the electronic supplementary material.

Supplementary Material 1

Supplementary Material 2

## Data Availability

No new individual patient data were generated in support of this research. All model parameters are reported within the article and additional supplementary materials.

## Footnotes

1 The ‘mean LCL’ here refers to the mean of all LCL determinations over the study period across all patients treated with cysteamine. It doesn’t refer to what we have called ‘annual LCL’ (i.e., the average LCL within an annum).

2 For example, one of the clinical experts consulted stated that in her experience it may be common for patients to be diagnosed before the age of 1. In this case we could assume that, given a CCS of 21.93, the representative patient has a treatment duration of 14.4 years and, thus, and ACS of 1.52.

3 In PSMs the survival probability in a health state at the start of a cycle is assumed to remain constant throughout that cycle, and only decreases at the start of the next cycle. This likely results in an overestimation of the actual time spent in that cycle. When considering the observed survival data from studies which inform PSMs, the survival probability is likely decreasing throughout any given ‘cycle’. A half cycle correction is the recalculation of the survival probability at the start of a cycle so that it is the average of the observed survival probabilities at the two timepoints which correspond to the start and end of the cycle. This adjustment often results in a more accurate reflection of the time spent in that cycle.

4 These proportions also correspond with clinical expert feedback stating that, in clinical practice in the United Kingdom (UK), most transplants are from living donors for nephropathic cystinosis patients. The UK Renal Registry [31] also provided data for all patients (adult and paediatric) showing 2441/3479 (~ 70%) of incident kidney transplant were from deceased donors in 109 – with similar proportions in 2018 and 2017. This alternate assumption was not considered, but in the sensitivity analyses (Table 6) the assumption of 100% deceased donors / 0% living donors and 0% deceased donors / 100% living donors were effectively considered by considering the extreme values for the waiting time for a transplant (0 or 24 months) and the time to transplant rejection/failure (17.5 or 22.5 years).

5 Of note, all results referred to in this and the next sections are per-patient total (lifetime) *discounted* results.

## Abbreviations

ACS: Annual Compliance Score
CCS: Composite Compliance Score
CTNS: Cystinosin, Lysosomal Cystine Transporter
GC: Good Compliance
HR: Hazard Ratio
IPD: Individual Patient Data
KF: Kidney Failure
KM: Kaplan-Meier
LCL: Leukocyte Cystine Level
NC: Nephropathic Cystinosis
NHS: National Schedule of National Health Service
NICE: National Institute of Health and Care Excellence
OS: Overall Survival
PSM: Partitioned Survival Model
PC: Poor Compliance
Q6H: Every 6 h
QOL: Quality-of-Life
SKF: Survival to Kidney Failure
UK: United Kingdom
