# Supplementary material for: Impact of compliance to oral cysteamine treatment on the costs of Kidney failure in patients with nephropathic cystinosis in the United Kingdom
Source: BMC Nephrol. 2023 Nov 29;24:351. doi: 10.1186/s12882-023-03392-y (PMC10688492; doi:10.1186/s12882-023-03392-y)
Supplement: Supplementary file 2 — Supplementary Material 2 [file 12882_2023_3392_MOESM2_ESM.docx]

**SUPPLEMENTARY TABLES AND FIGURES**

Contents

[Supplementary Figure 1: Parametric models fit to digitised data for SKF in the under-five group 1](#_Toc136851621)

[Supplementary Table 1: Goodness of fit for parametric curves (SKF in the under-five group) 1](#_Toc136851622)

[Supplementary Figure 2: Parametric models fit to digitised data for OS in the under-five group 2](#_Toc136851623)

[Supplementary Table 2: Goodness of fit for parametric curves (OS in the under-five group) 2](#_Toc136851624)

[Supplementary Figure 3: Parametric models fit to digitised data for OS in the OVER-five group 3](#_Toc136851625)

[Supplementary Table 3: Goodness of fit for parametric curves (OS in the over-five group) 4](#_Toc136851626)

[Supplementary Table 4: Healthcare resource group codes used for peritoneal dialysis cost calculation 4](#_Toc136851627)

[Supplementary Table 5: Healthcare resource group codes used for Haemodialysis cost calculation 4](#_Toc136851628)

[Supplementary Table 6: Results of micro costing calculations (assuming 3 transplants) 6](#_Toc136851629)

# Supplementary Figure 1: Parametric models fit to digitised data for SKF in the under-five group


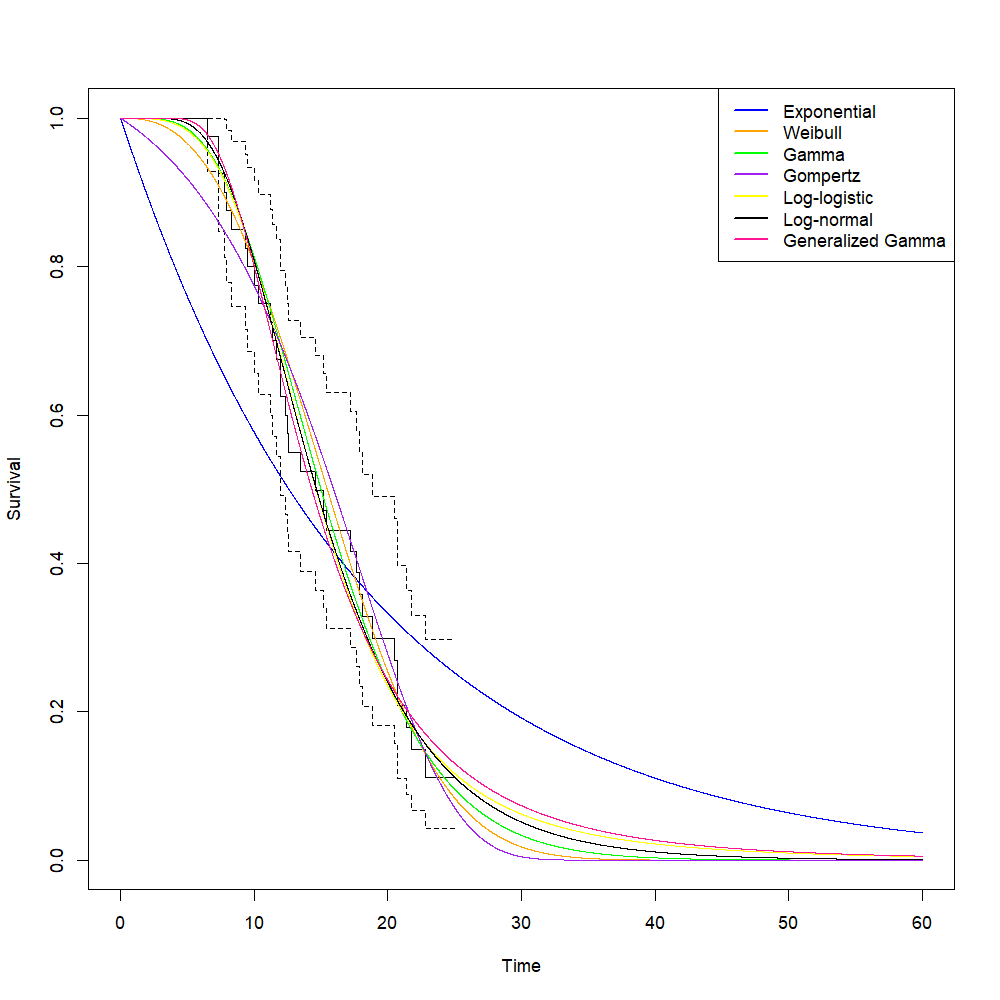


Note: the solid black line and dashed lines, missing from the key in the figure above, represent the digitised Kaplan-Meier data and the dotted 95% confidence intervals respectively.

# Supplementary Table 1: Goodness of fit for parametric curves (SKF in the under-five group)

| Model | AIC | BIC | AIC RANK | BIC RANK | COMBINED |
| --- | --- | --- | --- | --- | --- |
| Exponential | 259.337 | 261.025 | 7 | 7 | 7 |
| Weibull | 229.700 | 233.078 | 5 | 5 | 5 |
| Gamma | 226.978 | 230.356 | 2 | 2 | 2 |
| Gompertz | 236.260 | 239.637 | 6 | 6 | 6 |
| Log-logistic | 227.536 | 230.914 | 4 | 3 | 3 |
| **Log-normal** | **225.809** | **229.187** | **1** | **1** | **1** |
| Generalised Gamma | 227.378 | 232.445 | 3 | 4 | 3 |

AIC: Akaike information criteria, BIC: Bayesian information criteria

# Supplementary Figure 2: Parametric models fit to digitised data for OS in the under-five group


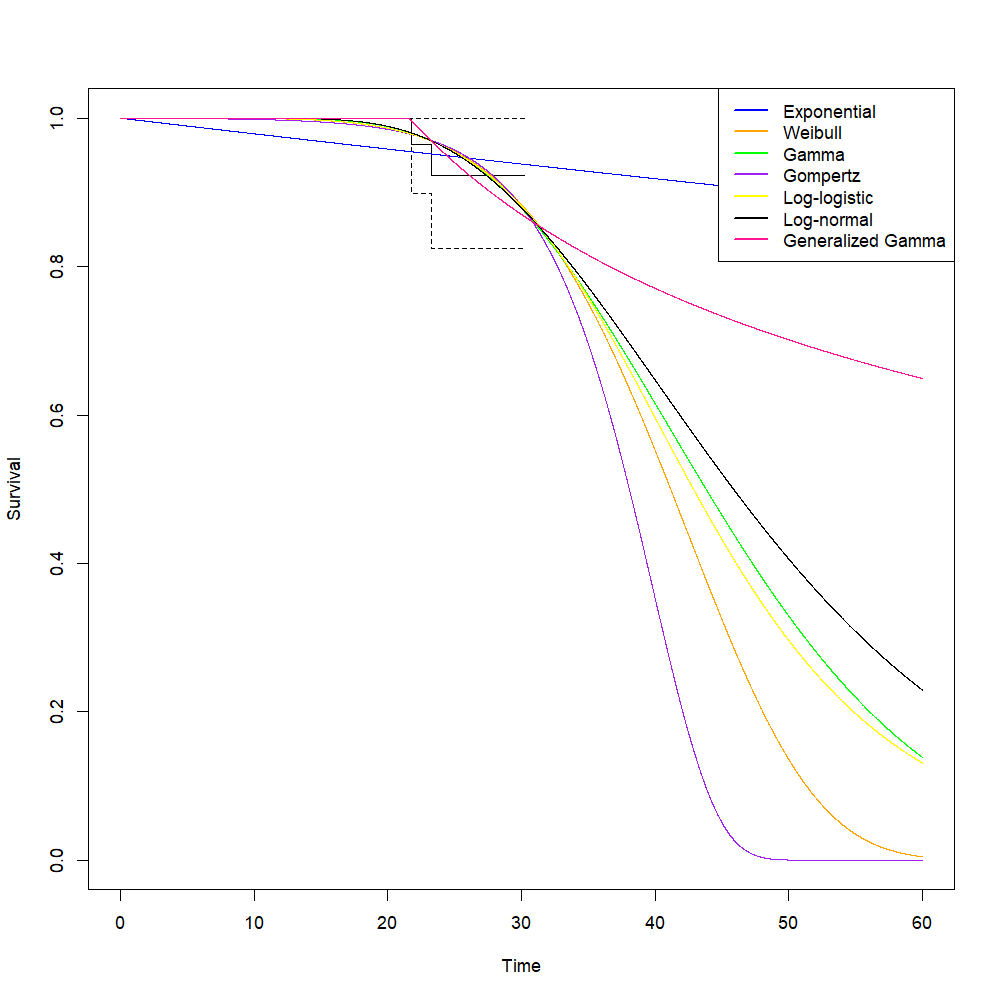


Note: the solid black line and dashed lines, missing from the key in the figure above, represent the digitised Kaplan-Meier data and the dotted 95% confidence intervals respectively.

# Supplementary Table 2: Goodness of fit for parametric curves (OS in the under-five group)

| Model | AIC | BIC | AIC RANK | BIC RANK | COMBINED |
| --- | --- | --- | --- | --- | --- |
| Exponential | 30.615 | 32.304 | 7 | 7 | 7 |
| Weibull | 28.285 | 31.662 | 5 | 5 | 5 |
| Gamma | 27.963 | 31.341 | 3 | 2 | 3 |
| Gompertz | 28.801 | 32.179 | 6 | 6 | 6 |
| Log-logistic | 28.224 | 31.602 | 4 | 4 | 4 |
| **Log-normal** | **27.815** | **31.192** | **2** | **1** | **1** |
| Generalised Gamma | 26.447 | 31.513 | 1 | 3 | 2 |

AIC: Akaike information criteria, BIC: Bayesian information criteria

# Supplementary Figure 3: Parametric models fit to digitised data for OS in the OVER-five group


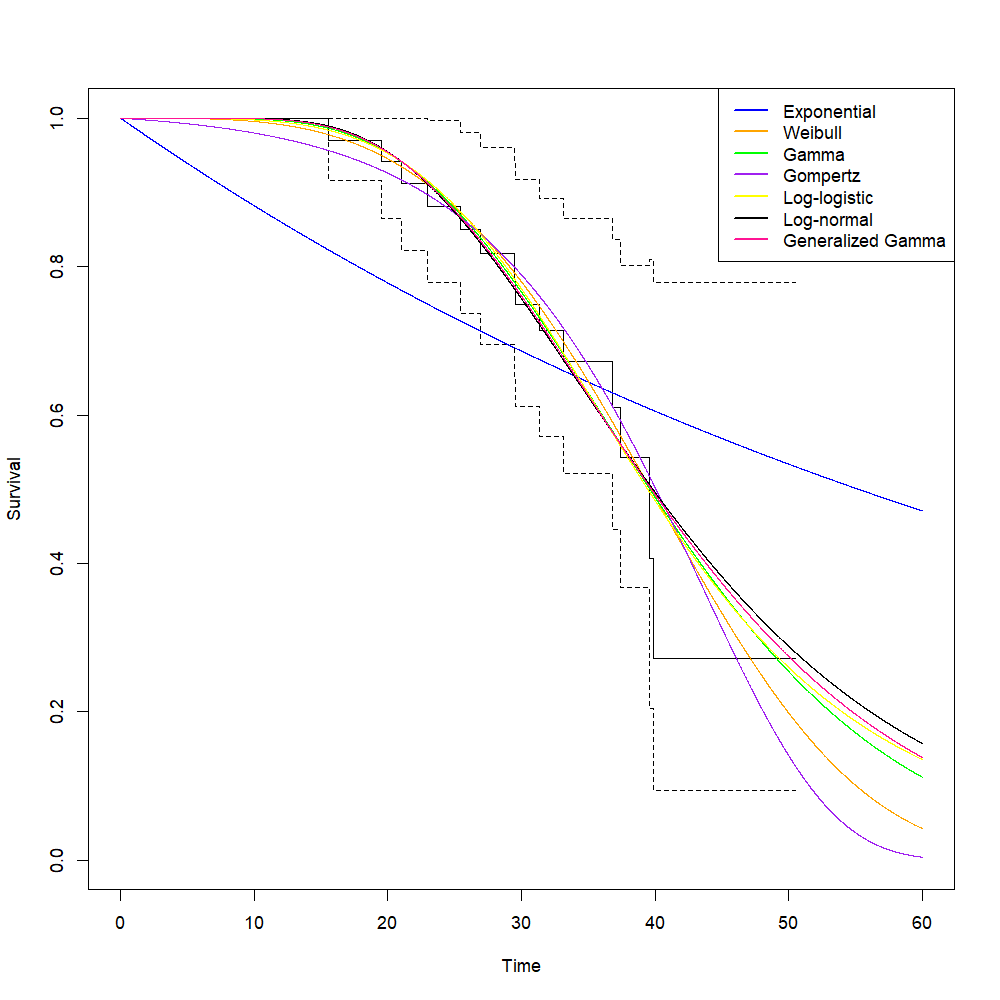


Note: the solid black line and dashed lines, missing from the key in the figure above, represent the digitised Kaplan-Meier data and the dotted 95% confidence intervals respectively.

# Supplementary Table 3: Goodness of fit for parametric curves (OS in the over-five group)

| Model | AIC | BIC | AIC RANK | BIC RANK | COMBINED |
| --- | --- | --- | --- | --- | --- |
| Exponential | 152.560 | 154.115 | 7 | 7 | 7 |
| Weibull | 134.489 | 137.599 | 4 | 4 | 4 |
| Gamma | 133.907 | 137.018 | 2 | 2 | 2 |
| Gompertz | 136.829 | 139.940 | 6 | 5 | 5 |
| Log-logistic | 134.039 | 137.150 | 3 | 3 | 3 |
| **Log-normal** | **133.888** | **136.999** | **1** | **1** | **1** |
| Generalised Gamma | 135.871 | 140.537 | 5 | 6 | 5 |

AIC: Akaike information criteria, BIC: Bayesian information criteria

# Supplementary Table 4: Healthcare resource group codes used for peritoneal dialysis cost calculation

| Currency | Currency Description | Activity | Unit Cost | Total Cost |
| --- | --- | --- | --- | --- |
| LD11A | Continuous Ambulatory Peritoneal Dialysis, 19 years and over | 335,625 | £76 | £25,514,294 |
| LD12A | Automated Peritoneal Dialysis, 19 years and over | 550,655 | £78 | £42,850,273 |
| LD13A | Assisted Automated Peritoneal Dialysis, 19 years and over | 134,361 | £89 | £11,976,154 |
| LD11B | Continuous Ambulatory Peritoneal Dialysis, 18 years and under | 8,830 | £133 | £1,172,806 |
| LD12B | Automated Peritoneal Dialysis, 18 years and under | 16,335 | £87 | £1,415,026 |
|  | **Weighted Average (per day cost)** | | | **£79.30** |
|  | **Per month cost *** | | | **£2,413.58** |

*Based on assumption that peritoneal dialysis is required daily. (1 month = [365.25/12])

Source: NHS Reference Costs 2019/20 [33]

# Supplementary Table 5: Healthcare resource group codes used for Haemodialysis cost calculation

| Currency | Currency Description | Activity | Unit Cost | Total Cost |
| --- | --- | --- | --- | --- |
| LD01A | Hospital Haemodialysis or Filtration, with Access via Haemodialysis Catheter, 19 years and over | 427,313 | £172 | £73,589,213 |
| LD02A | Hospital Haemodialysis or Filtration, with Access via Arteriovenous Fistula or Graft, 19 years and over | 669,071 | £166 | £111,314,122 |
| LD03A | Hospital Haemodialysis or Filtration, with Access via Haemodialysis Catheter, with Blood-Borne Virus, 19 years and over | 17,487 | £179 | £3,125,680 |
| LD04A | Hospital Haemodialysis or Filtration, with Access via Arteriovenous Fistula or Graft, with Blood-Borne Virus, 19 years and over | 21,497 | £181 | £3,899,618 |
| LD05A | Satellite Haemodialysis or Filtration, with Access via Haemodialysis Catheter, 19 years and over | 609,845 | £158 | £96,654,573 |
| LD06A | Satellite Haemodialysis or Filtration, with Access via Arteriovenous Fistula or Graft, 19 years and over | 1,111,887 | £160 | £178,296,656 |
| LD07A | Satellite Haemodialysis or Filtration, with Access via Haemodialysis Catheter, with Blood-Borne Virus, 19 years and over | 20,514 | £159 | £3,255,531 |
| LD08A | Satellite Haemodialysis or Filtration, with Access via Arteriovenous Fistula or Graft, with Blood-Borne Virus, 19 years and over | 45,043 | £166 | £7,467,326 |
| LD09A | Home Haemodialysis or Filtration, with Access via Haemodialysis Catheter, 19 years and over | 56,402 | £217 | £12,253,577 |
| LD10A | Home Haemodialysis or Filtration, with Access via Arteriovenous Fistula or Graft, 19 years and over | 83,737 | £180 | £15,094,559 |
| LD01B | Hospital Haemodialysis or Filtration, with Access via Haemodialysis Catheter, 18 years and under | 11,286 | £514 | £5,803,071 |
| LD04B | Hospital Haemodialysis or Filtration, with Access via Arteriovenous Fistula or Graft, with Blood-Borne Virus, 18 years and under | 171 | £942 | £161,059 |
| LD05B | Satellite Haemodialysis or Filtration, with Access via Haemodialysis Catheter, 18 years and under | 220 | £175 | £38,524 |
| LD06B | Satellite Haemodialysis or Filtration, with Access via Arteriovenous Fistula or Graft, 18 years and under | 234 | £282 | £66,004 |
| LD08B | Satellite Haemodialysis or Filtration, with Access via Arteriovenous Fistula or Graft, with Blood-Borne Virus, 18 years and under | 4 | £144 | £574 |
| LD09B | Home Haemodialysis or Filtration, with Access via Haemodialysis Catheter, 18 years and under | 1,515 | £281 | £425,064 |
| LD10B | Home Haemodialysis or Filtration, with Access via Arteriovenous Fistula or Graft, 18 years and under | 2,001 | £99 | £198,902 |
|  | **Weighted Average (per day cost)** | | | **£166.21** |
|  | **Per month cost *** | | | **£2,160.78** |

*Based on assumption that haemodialysis dialysis is required 3 times/7days. (1 month = [365.25/12])

Source: NHS Reference Costs 2019/20 [33]

# Supplementary Table 6: Results of micro costing calculations (assuming 3 transplants)

| Stage | Time spent (months) in each stage: unadjusted | Time spent (months) in each stage: adjusted by probability of survival during that stage | Annual Cost | Lifetime cost | | |
| --- | --- | --- | --- | --- | --- | --- |
|  |  |  |  | Good Compliance | Poor Compliance | Difference |
| Pre-transplant dialysis^†^ | 9.04 | 8.91 | £585.33 | £5,234 | £6,677 | -£1,443 |
| First transplant^‡^ | NA | 0.98 | £835.82 | £7,474 | £9,534 | -£2,060 |
| Post first transplant maintenance^§^ | 247.40 | 180.47 | £561.41 | £5,020 | £6,404 | -£1,384 |
| First failed transplant dialysis^†^ | 9.04 | 4.70 | £308.87 | £2,762 | £3,523 | -£761 |
| Second transplant^‡^ | NA | 0.52 | £441.04 | £3,944 | £5,031 | -£1,087 |
| Post second transplant maintenance^§^ | 247.40 | 95.23 | £296.25 | £2,649 | £3,379 | -£730 |
| Second failed transplant dialysis^†^ | 9.04 | 2.48 | £162.98 | £1,457 | £1,859 | -£402 |
| Third transplant^‡^ | NA | 0.27 | £232.73 | £2,081 | £2,655 | -£574 |
| Post third transplant maintenance^§^ | 247.40 | 50.40 | £156.81 | £1,402 | £1,789 | -£387 |
| Third failed transplant dialysis^¶^ | NA | 58.27 | £3,827.38 | £34,225 | £43,659 | -£9,434 |
| Total | NA | 400.46^#^ | £7,408.62 | £66,250 | £84,510 | -£18,261 |

KF: Kidney Failure, NA; Not Applicable, NHS: National Health service.

* The probability of survival per month within the post KF state was estimated by assuming an exponential distribution of survival probabilities based on the mean time spent in the post-KF state (i.e., 33.37 years; equivalent to ~400.46 months). Costs were calculated by considering an average patient’s pathway through the consecutive stages. I.e., first through the “pre-transplant dialysis” (waiting list) phase, and then into their first transplant, and then into the maintenance (time to transplant rejection) phase and so on. The amount of time assigned to each stage was taken from real world data (2^nd^ column) subsequently adjusted by the probabilities of survival during the relevant stage (3^rd^ column), and then multiplied by the relevant cost associated with that phase (4^th^ column).

† The time spent in this stage is based on the time spent on the waiting list for a transplant (Table 4). The unit cost applied here is the monthly cost of dialysis (Table 4), £ 2,192.12.

‡ The value in the 3^rd^ column of this row does not represent the *time* spent undergoing a transplant (this unspecified time is assumed to be absorbed within the subsequent post, transplant stage). Rather it represents the probability of undergoing a *transplant at that time*. This probability is applied to the full annual cost of a kidney transplant in the first year (Table 4), £ 28,526.52.

§ The time spent in this stage is based on the time to transplant rejection or failure (Table 4). It is provided in years (20.62) in Table 4 and converted to months (247.40) here. The unit cost applied here is the subsequent monthly cost of kidney transplant (i.e., maintenance costs), £ 110.93, (Table 4). The unit cost in the first transplant maintenance stage is multiplied by 168.90 months, not 180.47 months, because the cost in the initial 12-month period in this stage (11.57 months accounting for survival probabilities) is accounted for by the annual cost of a transplant. Similarly, the unit cost in the second transplant maintenance stage is multiplied by 89.12 not 95.23 because the cost in the initial 12-month period (6.11 months accounting for survival probabilities) is accounted for by the annual cost of a transplant. The unit cost in the third transplant maintenance stage is multiplied by 47.18 not 50.40 for the same reason.

¶ As the average patient undergoes 3 transplants, the time spent in this stage is the remaining time in the post KF state post rejection/failure of the 3^rd^ transplant. Therefore, there is no “unadjusted” value here - the number of months spent in this stage must be directly calculated.

# This sum does not account for the values in the “first transplant”, “second transplant”, or “third transplant” rows as these values represent probabilities rather than time.
